# Exploring the potential impact of health promotion videos as a low cost intervention to reduce health inequalities: a pilot before and after study on Bangladeshis in inner-city London

**DOI:** 10.1080/17571472.2016.1208382

**Published:** 2016-08-03

**Authors:** Suleman Latif, Imad Ahmed, Mohammed Shafiul Amin, Ismail Syed, Na’eem Ahmed

**Affiliations:** ^a^ Norfolk and Norwich University Hospitals NHS Foundation Trust, Norwich, UK; ^b^ West Hertfordshire Hospitals NHS Trust, West Hertfordshire, UK; ^c^ Medway NHS Foundation Trust, Kent, UK; ^d^ East Lancashire Hospitals NHS Trust, Lancashire, UK; ^e^ St George’s University Hospitals NHS Foundation Trust, London, UK

**Keywords:** Coronary disease/etiology, health services need and demand, instructional films and video, health promotion, education of patients

## Abstract

**Background:**

Coronary artery disease is the single most common cause of death in the UK. For those born in Bangladesh but dying in England and Wales, coronary artery disease causes 25% of all deaths. Cost-effective solutions are required to address this burden. Several studies have demonstrated the effectiveness of educational video intervention in informing patients in various settings.

**Setting:**

A Bangladeshi women’s group in South London.

**Questions:**

The effectiveness of a health educational video in influencing the knowledge and attitudes towards a preventable illness amongst Bangladeshis in London? The scope of videos for health promotion?

**Methods:**

An educational video on the signs, symptoms and prevention of coronary artery disease was played to a Bangladeshi women’s group in South London in the Bengali language. Participants (*n* = 18, mean age = 53.7) completed a fifteen-question survey to assess their baseline knowledge prior to viewing (pre-test). The group then viewed the video and repeated the initial questionnaire, with additional questions to solicit their attitudes and perceptions (post-test).

**Results:**

The intervention significantly improved the basic knowledge of coronary artery disease. There was a statistically significant improvement in the number of correct responses amongst participants with *p* = 0.0002 (mean change 2.28, 95% CI 1.29–3.27) and in the number of unsure responses *p* = 0.0042 (mean change 1.83, 95% CI 0.01–3.01). Upon viewing the video, all participants agreed that they wanted to implement the advice from the video into their current lifestyles.

**Conclusion/Discussion:**

The educational video significantly improved the knowledge and attitudes pertaining to coronary artery disease amongst British-Bangladeshi individuals in the UK community setting. This project illustrated how commissioners may effectively utilise third-sector organisations through partnerships to implement innovative methods of health screening and promotion. Videos are a novel approach of providing culturally sensitive health education to ethnic minority groups, through screening in clinics and in local media.

## Why this matters to me

I am currently a core medical trainee, having qualified from medical school three years ago. However, this project began as a medical student during my final years of study through volunteering with like-minded colleagues. Through our experiences at medical school and the patient demographic we interacted with in South London, we became very aware of the burden of non-communicable, chronic diseases amongst the patient population we dealt with. Anecdotally, we found that the problems were certainly not uniform across the age, ethnic and socioeconomic spectrum.

On a personal level, my own parents are of the diaspora community from South East Asia of which this work relates to. I have seen first hand in this community the issues with regards to uptake of health services. This – as well as review of literature – has led me to believe that as providers of healthcare, we must use novel approaches to bridge gaps and reduce inequalities in health. In an age of progressive information technologies and social networking, educational videos are a current and relevant approach, which may be utilised to implement change. This study also illustrates the potential for the employment of voluntary bodies for addressing some of the more pressing issues in the current healthcare context.

## Key message(s)

• Health promotional videos have potential in addressing health inequalities.
• Commissioners and consortia can effectively utilise third sector organisations to gather data, and to find innovative ways to carry out health screening and promotion.
• Educational videos, such as the one used in this study, are possibly an economically viable intervention tool for educating the Bangladeshi community in the UK, but also other ethnic minorities.
• There is certainly a need for further research into the optimal use of video intervention in providing health education, with respect to the setting, content and timing.

## Introduction

### Background

Coronary artery disease is the single most common cause of death in the UK, accountable for one in every five male deaths and one in every eight female deaths in 2008.[1] Further highlighting its impact is that it has also been established as the most common reason for premature deaths in the UK (below age 75). Though such statistics are alarming, the impact of coronary heart disease has been on the decline over the past few decades and it has since been suggested that secondary prevention and treatment measures can explain approximately two thirds of the improvement, suggestive of the potential for interventional prevention measures.[2]

There clearly exists significant variation of the impact of coronary heart disease amongst inhabitants of the UK, owing largely to demographic differences. As mentioned, although the UK as a whole has demonstrated a decline in mortality rates for coronary heart disease, there exists rising trends internationally, notably in South East Asia.[3] Amongst the factors proposed to explain the rising trend in cardiovascular diseases in Bangladesh, for example, is the widespread lack of awareness of healthy behaviour patterns.[4] It seems that migration factors have led to transference of the burden of coronary artery disease. For those born in Bangladesh but dying in England and Wales, coronary artery disease causes 25% of all deaths, whereas for those born in the UK, it is responsible for 15% of all deaths.[5]

Access to education on heart disease is provided in various modalities but the key issue is arguably in its take-up, especially amongst non-English speaking communities that have been shown to shoulder a relatively higher burden of coronary artery disease. Cost-effective and engaging solutions are required to enhance their awareness of the risk factors and preventative measures for this disease.

### The role of video intervention

Several studies have demonstrated the effectiveness of health educational video intervention as a novel way of informing patients in various settings. In the inner-city tertiary care setting in the US, an emergency department waiting area was utilised to effectively disseminate video-based information on coronary artery disease.[6] It is well established that video is an effective strategy of health information dissemination in the developing world.[7] More recently, a similar setting in India was used to inform patients on myocardial infarction, demonstrating short-term benefits.[8]

### Goals of this study

Video intervention has not yet (at the time of writing) been studied to assess the effects on an immigrant population to a developed country that strongly adheres to cultural norms – such as a Bangladeshi community in inner city London in the first language of that population. This study aims to address this by assessing the potential effectiveness of video education for addressing major public health issues such as coronary artery disease, its modifiable risk factors and practical guidance for viewers in the community setting amongst Bangladeshi immigrants to the UK. We wished to explore the influence that an educational video on coronary artery disease, its risk factors and prevention, would have on patients’ knowledge and their attitudes towards their lifestyles through this pilot study on a small sample population.

### Aim

To study the use and effectiveness of video health promotion for a preventable disease – coronary artery disease – in the community setting, amongst an immigrant sample population of eighteen in London, delivered in their first language. To also provide further insight into this method of health promotion in this context, which to date has not been significantly explored from the evidence of current, available literature.

## Method

### Study type

This is a before-and-after pilot study to assess the effectiveness of the video intervention in impacting on viewers’ knowledge and attitudes in regards to coronary artery disease, with a view to shed more light into this mode of health promotion.

### Study site and participants

The study was implemented in the London borough of Southwark. Southwark has a population of over 250,000, with a small South Asian community (4%). Circulatory disease accounts for 34% of hospital admissions in Southwark, within Bangladeshi adults however, it accounts for a higher proportion of admissions (51%).[9] We selected Rockingham Centre, a community centre in Elephant and Castle, Southwark, which holds regular women’s groups attended primarily by the local Bangladeshi women of the surrounding council estates. The inclusion criteria from the women’s groups were those aged 18 years or greater, born in Bangladesh and having Bengali as their first language. Those born in a country other than Bangladesh and non-consenting individuals were not included in the study.

### Survey questionnaire

All participants (*n* = 18, mean age = 53.7) completed a fifteen-question survey to assess their baseline knowledge prior to viewing (pre-test). All participants then viewed the video delivered in Bengali pertaining to issues of coronary heart disease. Finally, the initial questionnaire was repeated, with an additional four questions. The pre-test consisted of 15 non-validated questions – 5 questions assessing the basic knowledge of coronary artery disease and 10 questions related to risk factors for cardiovascular disease. Each question invited the answers of ‘True’, ‘False’ and ‘Don’t know’. The post-test contained all of the questions of the pre-test but with four additional questions to solicit their attitudes and perceptions towards the video as an educational tool and also to elucidate views on their lifestyles.

### The interventional video

An educational video was produced on coronary artery disease in the Bengali language. The subject matter was selected for its current prevalence, and the added magnitude amongst the Bangladeshi population both in the UK and in Bangladesh. Furthermore, the topic would be potentially beneficial to both genders, is relevant or familiar to most viewers and is not viewed as taboo as perhaps some other health related issues are amongst this demographic.

The video contained information in concordance with the guidelines promoted by British Heart Foundation, but with a focus towards the Bangladeshi culture. In the video, a UK based consultant explains – in Bengali – basic heart physiology, coronary artery disease and its modifiable risk factors with practical guidance for viewers. The video was shot in an interview style with the use of a heart model and diagrams to illustrate basic heart physiology. The video was just over eight minutes in length.

Efforts were made to ensure that the content of the video was fully comprehensible for the targeted viewing audience, given the apparent imbalance in medical diction that exists between the English and Bengali language. The content of the video was verified by two Bengali speaking individuals and further by a Bengali-speaking communications specialist of the NHS.

### Survey administration

Once the group for inclusion had been clearly identified, the pre-test survey was administered. A Bengali-speaking general practitioner was required to read aloud the pre-test and also the post-test questionnaires to the illiterate participants present. The interventional video was screened in a single sitting to all participants of the study. The post-test was administered immediately after the video screening. The research team clearly instructed all participants that there should be no collaboration attempted with other participants or with the research team themselves.

Upon completion of the post-test, all surveys were collected from the participants and the general practitioner present explained the correct answers to all questions to the participants.

### Data analysis

Dependent sample *t*-tests were implemented to assess the statistical significance in the differences of the mean numbers of responses of the participants from the pre-test and post-test data. All statistical data calculations were obtained by Microsoft Excel software. Differences were deemed statistically significant at *p* < 0.05.

## Results

Eighteen participants completed the sequence of pre-test – viewing – post-test for the benefit of this study. Mean age was 53.7 (SD 13.4, range 18–72). All were women born in Bangladesh with Bengali as their first language.

Prior to the screening at baseline (pre-test), the mean number of correct responses from the fifteen survey questions was 9.38 (SD 1.97). The post-test survey demonstrated results of a mean score of 11.67 (SD 1.50). The difference of the means for the group was found to be statistically significant, giving a p-value of value of *p* = 0.0002 (mean change 2.28, 95% CI 1.29–3.27). The effect of the intervention was deemed large and the means are likely very different given the Cohen’s *d* value of 1.14 and a coefficient of determination of 0.58.

There was also a significant reduction in the number of unsure responses from the pretest to the post-test, giving *p* = 0.0042 (mean change 1.83, 95% CI 0.01–3.01).

## Discussion

The study hypothesis was that the educational video on coronary artery disease, its risk factors and prevention would significantly improve patients’ knowledge in the short term, amongst an immigrant population in their first language – Bengali. The data analysis of our study showed that the video on issues pertaining to coronary artery disease and its prevention was indeed significantly effective. Specifically, this was shown by the statistically significant improvement in the responses elicited from the participants in surveys administered before and after the viewing of the film. Both the improvement in correct responses and the decline in unsure responses firmly support the positive impact of the intervention.

This suggests that (a) an educational video is an effective means of improving the knowledge of viewers in the short term and (b) this educational video was of adequate quality and content to impact on the knowledge and attitudes of its viewers.

All participants agreed or strongly agreed that they would apply the advice given in the video to their current lifestyles. Sixteen of the eighteen agreed or strongly agreed to learning something new about heart disease from watching the video. These were, albeit, responses taken immediately after viewing of a health promotional film and may pertain to the viewers positive response to the film itself rather than the changing effect it had or will continue to have on them. It is, perhaps, unrealistic to assume that the eight-minute viewing will considerably change their lifestyle practically in line with their survey response. The revelation that almost all respondents learnt something new from the video underlines the knowledge void that exists on basic health matters amongst this demographic of the population and the potential for accessible, informative solutions.

As a development to this pilot study, a similar but larger cohort study with a post intervention survey at an interval greater than a month would provide an insight into the longer-term effectiveness of the intervention video, and also shed light on the sustainability of the changes in attitudes and lifestyles of the participants that have provisionally been perceived in this study. The possibility of the influence of a primacy effect on the responses offered by participants due to the timing of the pre and post intervention surveys relative to the timing of the screening would also be addressed by such a follow-up.

The screening of the health educational film was held in a community centre setting and such videos could possibly be played in highly concentrated regions of the Bangladeshi population, in clinics and waiting rooms. An informed patient prior to consultation makes for a more productive patient–doctor liaison, particularly when doctors may be time-constrained and consequently unable to offer similar information effectively. Given the poor uptake of health services in this community, it may benefit from follow-up educational provision.

The finding that the educational video on coronary artery disease was effective in a sample population where the disease is especially problematic serves as provisional evidence to suggest that similar videos on other preventable diseases can be produced and utilised to educate selected demographic groups in the UK. This may include areas of health typically attached with stigma, which to an extent explain poor access to services amongst certain groups in the population, such as that in mental and sexual health. The impact of such videos could be extended to other ethnic minority groups and also to the countries of origin – in this case, Bangladesh – where the health burdens are particularly great.

## Limitations

This pilot study has certain limitations that present areas of development to further research. A screening questionnaire on the participants’ health and educational background would be particularly useful, with the possibility that those of a greater level of education or with heart related health issues being more likely to perform better than others on the administered surveys. The selection of participants, the non-randomised design of the study and also the lack of a control group for comparison are the primary methodological areas that could be addresses in future work in addition to longer term follow-up surveys as previously mentioned. Efforts taken to address these issues for future studies would further clarify the level of effectiveness of health education videos for influencing viewers’ awareness and choices.

Although the administration of identical pre-test and post-test surveys provided a homogenous means of performance measure, this may have presented an area of unreliability if participants used the film to focus on the content that was to be assessed, given that they were at that instance aware of the pre-test questions. Furthermore, due to the largely illiterate sample group, the reading aloud of the survey questions and subsequent eliciting of responses was unavoidable. The vocalisation of the surveys may have introduced bias through tone, although all efforts were made by the research team to avoid influence of the participants in any way. The issue of illiteracy amongst this demographic group is one of the key reasons why health information leaflets are deemed ineffective for many, as well as issues of accessibility despite their availability in several languages – issues that were uncovered anecdotally through conversation with diaspora communities and used as a basis for searching for alternative, novel methods of health promotion.

This pilot study did not financially assess the cost-effectiveness of the use of videos as a health promotional tool on a wider scale, nor was that the purpose. In the current system of commissioning, where general practitioners have direct control of local budgets and referral pathways, new cost-effective ways of promoting healthier lifestyles and disease prevention may need to be considered. The aim here was to shed light on a novel method of health promotion that has not yet been explored and exploited to any significant extent. Further developed studies aimed at providing conclusive data on how specific health promotional tools can contribute to public health policy, would certainly require a thorough cost-benefit analysis.

## Conclusion

We found that a video of just over eight minutes in length on issues pertaining to coronary artery disease in the Bengali language significantly improved the knowledge and attitudes of viewers in the community setting in inner-city London. The effect was calculated to be large, demonstrated by the improvement in correct response and the decline of unsure responses amongst the participant group, consisting of UK inhabitants of Bangladeshi origin. This provides an illustration of the potential of health promotional videos in addressing health inequalities.

The implications in practice are vast. This study illustrates the potential for the utilisation of voluntary bodies for addressing some of the more pressing issues in the current healthcare context. In an economic climate in which health service providers – including academic organisations – are actively seeking measures to reduce costs, commissioners and consortia can effectively utilise third sector organisations to gather data, and to find innovative ways to carry out health screening and promotion. The ability of voluntary organisations in this role can be used to augment and compliment the work that academic organisations do to collect data and find new cost effective ways to promote healthier lifestyles and screen for preventable disease.

Educational videos, such as the one used in this study, are possibly an economically viable intervention tool for educating the Bangladeshi community in the UK, but also other ethnic minorities. This is pertinent, given the issues of access to healthcare education compounded by the existence of cultural stigma associated with certain aspects of health amongst these communities. There is also replication potential in Bangladesh, where these videos can be made accessible in clinics and in the media. There is certainly a need for further research into the optimal use of video intervention in providing health education, with respect to the setting, content and timing.

## Disclosures and conflicts of interest

NA heads Selfless, an international development organisation that works to address inequalities in health and education. There are no financial competing interests to disclose.

## Appendices

Appendix Table A1. Questions 1–15 of the pre-test and post-test surveys.

**Table:**

	True	False	Don’t Know
1. The heart works like a pump			
2. Blood vessels can get blocked			
3. Heart attack can lead to permanent heart damage			
4. Breathlessness might be due to heart problems			
5. Diabetes and heart disease are connected			

The following INCREASES risk of cardiovascular disease:

**Table:**

	True	False	Don’t Know
6. Smoking cigarettes			
7. Weight loss			
8. Obesity			
9. Hypertension			
10. Depression			
11. High fatty diet			
12. Family history of heart disease			
13. Daily exercise			
14. Sleeping too much			
15. Diabetes			

Appendix Table A2. Post-test participant perspective questions.

**Table:**

	Strongly agree	Agree	Neutral	Disagree	Strongly disagree
I found the information on the video useful					
I learnt something new about heart disease					
I would watch the video again if I could					
I want to apply the advise from the video to my current lifestyle					
